# Evaluation of directly observed treatment for tuberculosis in the Bojanala health district, North West Province of South Africa

**DOI:** 10.4102/phcfm.v3i1.191

**Published:** 2011-03-14

**Authors:** John M. Tumbo, Gboyega A. Ogunbanjo

**Affiliations:** 1 Department of Family Medicine and Primary Health Care, University of Limpopo, Medunsa campus, South Africa

## Abstract

**Background:**

Tuberculosis (TB) remains one of the top public health problems in South Africa. Approximately 150 000 new cases and 10 000 TB-related deaths are reported in South Africa annually. In declaring TB a global emergency in 1993, the World Health Organization developed control strategies that include active case finding, laboratory support, directly observed treatment (DOT), contact tracing, and prevention of multidrug– and extreme drug-resistant tuberculosis (MDR-TB and XDR-TB). High DOT rates reported in some countries have been discordant with ‘low cure’ and ‘high MDR’ rates.

**Objectives:**

The aim of the study was to evaluate the use of DOT for TB in the Bojanala health district, North West Province, South Africa, by estimating the proportion of DOT use (1) amongst all TB patients and (2) in the initial TB treatment regimen compared to retreatment regimens.

**Method:**

A cross-sectional, descriptive study was conducted in 2008. Data regarding implementation of DOT were collected from eight purposefully selected primary health care clinics and one prison clinic in the health district. Upon receiving their informed consent, a questionnaire was administered to patients receiving TB treatment at the selected facilities.

**Results:**

A total of 88 (of 90 selected) patients participated in the study, of whom 50 (56.8%) were on DOT and had DOT supporters. However, 35 (40%) had never heard of DOT. DOT was used mainly for patients on the retreatment regimen (87.5%), rather than for those on first-line treatment (48.6%).

**Conclusion:**

In this South African rural health district, the DOT utilisation rate for TB was 56.8%, mainly for patients on the TB retreatment regimen. Strict implementation of DOT in all patients undergoing TB treatment is a known strategy for improving TB cure rate and preventing recurrence and drug resistance.

## Introduction

Tuberculosis (TB) remains the top infectious cause of mortality in adults, with an estimated 1.7 million deaths globally in 2004 and 1.8 million in 2008. Almost a third of the world's population (more than 2 billion people) are infected with the TB bacilli, which cause TB, and one in every 10 infected people is estimated to become sick with active TB in their lifetime.^1^ TB is a major public health problem in South Africa, with the country recently having been ranked fifth on the list of 22 high-burden TB countries in the world. According to the Global TB Report 2009 of the World Health Organization (WHO), South Africa had nearly 460 000 new cases in 2007, with an incidence rate of an estimated 948 cases per 100 000 population – a major increase from 338 cases per 100 000 population in 1998.^2^

According to the Department of Health's national TB management and control programme, South Africa had 341 165 TB cases in 2006, of which 131 100 were new smear-positive cases, 42 751 were smear-negative cases, 72 276 were no-smear cases and 40 431 were retreatment smear-positive cases. The incidence of all TB cases in the same year was 720 per 100 000.

Such high infection rates have been compounded by the HIV and AIDS epidemic, especially in sub-Saharan Africa where TB is the most common opportunistic infection in HIV-infected individuals. It is estimated that 73% of new active TB cases are associated with HIV infection.^3^ 1n 1991, targets set for national TB control programmes at the WHO's World Health Assembly aimed to detect at least 70% of all new sputum smear-positive cases arising per year and cure at least 85% of them by the turn of the century.^4^ The DOT short course (DOTS) is widely promoted as the most cost-effective strategy for controlling TB and has proved very effective in countries where it has been correctly implemented.^5^ Examples of successful DOT implementation have been reported in Cambodia and China, where as many as 84%–95% and 93%–97% of patients, respectively, were successfully treated between 1994 and 2003.^6^ The critical component of the DOTS strategy is the direct observation of treatment.

However, in some countries and communities, low cure rates and high rates of multidrug-resistant TB (MDR-TB) are discordant with the high levels of DOTS coverage recorded. For example, in South Africa the cure rates of smear-positive cases rose by only 13.2% between 2004 and 2007 (50.8% vs 64.0%), although DOTS coverage was reported to be 100%.^7^ The Bojanala health district in the North West Province of South Africa is one example: in 2007 the TB cure rate was only 47.9% in an area that recorded 100% DOTS coverage.^8^ This discrepancy was the motivation for conducting a study to understand the dynamics of DOT implementation in this health district.

## Ethical considerations

Ethical approval for the study was obtained from the Medunsa Research Ethics Committee, University of Limpopo (MCREC/M/17/2007: IR). Written and signed informed consent was obtained from all participants. Confidentiality was maintained during the study.

## Method

The aim of the study was to evaluate the use of DOT for TB in the Bojanala health district, North West Province, South Africa. A cross-sectional, descriptive study was conducted in 2008. At the time the Bojanala district had an estimated population of 1 185 325 and 117 primary health care facilities.

The study population consisted of patients from 52 primary health care facilities in two subdistricts where high DOT rates associated with low cure rates were recorded. Eight government primary health care clinics with the highest number of TB patients and one prison clinic were purposefully selected. A semi-structured questionnaire was administered individually to 10 consecutive adult TB patients at the respective clinics on a convenient day. Data were captured and analysed in Microsoft Excel.

## Results

Eighty-eight patients participated in the study, of whom 80 were from government primary health care clinics and the remaining eight from the prison clinic (two inmates refused to take part after selection). This translated to a response rate of 97.8%.

Adherence to TB treatment was reported to be 91%, with only 4.5% having missed treatment for more than 7 days in the preceding month. Slightly more than half of the respondents (56.8%) reported having a DOT supporter, and 54.5% who reported to be on DOT had been ‘consistently observed’ taking their TB treatment. More than a third of respondents (38.6%) reported that they ‘were not observed’ taking their TB treatment by a DOT supporter, especially if they were not on DOT, whilst 6.8% were irregularly observed by a DOT supporter. The majority (72%) of DOT supporters were health care professionals and only 24% were patients’ family members. For 80% of respondents DOT occurred at health care facilities, whilst 16% were observed at home and 4% at other sites (Table 1).

**TABLE 1:** Characteristics of the Tuberculosis patients.

Parameter	Characteristic	Responses	

		*N*	%
Patient awareness of DOT	Yes	53	60.2
	No	35	39.8
Patients with DOT supporters	Yes	50	56.8
	No	38	43.2
TB treatment regimen	Regimen 1†	74	84.1
	Regimen 2‡ (retreatment)	14	15.9
Adherence to TB treatment	Have not missed treatment	80	91.0
	Missed 2–6 days in the preceding month	4	4.5
	Missed more than 7 days in the preceding month	4	4.5
DOT supporter (*n* = 50)	Health care professional	36	72.0
	Family member	12	24.0
	Lay DOT supporter appointed by NGO	2	4.0
Observation of treatment	Consistently	48	54.5
	Irregularly	6	6.8
	Not observed at all	34	38.6
Site for observation (*n* = 50)	Clinic or hospital	40	80.0
	Home	8	16.0
	Elsewhere	2	4.0

† Regimen 1 includes rifampicin, isoniazid, ethambutol, and pyrazinamide to treat first occurrence

‡ Regimen 2 includes rifampicin, isoniazid, ethambutol, pyrazinamid and streptomycin to treat recurrence, re-infection and reactivation; *N*, number of patients responses.

For patients on treatment regimen 1, 40.9% were on DOT, whilst 43.2% were not. For patients on treatment regimen 2, 13.6% were on DOT, whilst 2.3% were not. Results were significant at the 0.05 decision level (*χ*^2^ = 5.11, *p* = 0.024 as calculated according to the Yates method shown is table 2).

**TABLE 2:** Treatment regimen and directly observed treatment use.

Treatment	TB Patients					

	Yes, DOT		No, DOT		Total	
		
	*N*	%	*N*	%	*N*	%
Regimen 1	36	40.9	38	43.2	74	84.1
Regimen 2	12	13.6	2	2.3	14	15.9

**Total**	**48**	**54.5**	**40**	**45.5**	**88**	**100**

Regimen 1 includes rifampicin, isoniazid, ethambutol, and pyrazinamide to treat first occurrence; Regimen 2 includes rifampicin, isoniazid, ethambutol, pyrazinamide and streptomycin to treat recurrence, re-infection and reactivation; DOT, directly observed treatment; *N*, number of patients.

## Discussion

DOTS is the internationally recommended strategy for TB control. It has five components, namely, (1) active case finding, (2) laboratory support, (3) treatment using DOT, (4) contact tracing and (5) prevention of MDR-TB and XDR- TB. DOTS coverage is the percentage of the population that falls within the administrative areas where the DOTS control strategy is used.^4^ This arbitrary definition likely accounts for the assertion that DOTS coverage is 100% in South Africa. DOTS coverage in South Africa increased from 22% in 1998 to 100% in 2006.^7^

At the core of the DOTS strategy is the direct observation of treatment, which entails a short course of chemotherapy for TB given under direct observation. A health care worker, family member or other trained person watches and helps as the patient swallows each dose of anti-TB medication. The rate of DOT implementation is a major determinant of the success of the DOTS strategy.

The main finding of this study was that DOT for TB was used by only 54.5% of the patients. This rate falls far below the 100% goal set by both the South African TB Control programme and the national TB control strategic plan.^9, 10^ The low implementation likely contributes to the low TB cure rate (47.9%) reported for this district in 2007— a proportion that fails to meet the national target of 85%.^8^ If the observed DOT implementation rate were to be extrapolated nationally, it would retard progress towards the country's attaining the United Nations’ Millennium Development Goal 6, which describes a global plan to reduce TB prevalence by 50% by 2015 and elimination by 2050.^11^

In this study DOT was used mainly by patients on treatment regimen 2. This goes against the principle of treating TB correctly the first time. It is from this pool of patients that the emergence of MDR-TB and XDR-TB are usually reported.

The place of observation and the responsible person are important factors for successful DOT implementation. In this study DOT was mainly administered at the health care facilities rather than at patients’ homes. The study also found that DOT was mainly administered by health care professionals (72%) rather than by lay DOT supporters. In the Bojanala district, many lay DOT supporters are employed by non-government organisations. However, the value of their involvement has not been realised as there is no formal structure linking them with mainstream health service delivery. Randomised controlled trials conducted in South Africa have shown that TB patients supported by lay DOT supporters can comply to their treatments just as well as those supported by health care professionals.^12^ Professional DOT support entails challenges of access, cost of travel and lack of health care personnel to provide a service that should ideally be community based.^13^ This mode of DOT support possibly contributes to high defaulter rates and low cure of TB in the district. An important observation of this study is the possibility of misreporting DOTS, which, by being a national policy, may therefore be accepted as policy in each administrative district in South Africa regardless of actual implementation.

### Study limitations

Although the sample frame and size may be considered a study limitation, efforts were made to have a fair representation of the TB patients in the health district. Another limitation was that the study focused only on one component of the DOTS strategy and did not evaluate the effects of other factors (e.g. HIV infection, poverty, political will and changing characteristics of the disease) that contribute to the TB epidemic.

## Conclusion

This study found that the use of DOT in this rural health district was much lower than the national target. The DOT utilisation rate of 54.5% was consistent with the reported low TB cure rate of 47.5%, although a direct causal relationship could not be proved. DOT was used mainly by patients on the retreatment regimen rather than by those on first-time treatment. It is recommended that all TB patients should be on DOT and more emphasis should be on the use of DOT at first diagnosis.
